# Iowa Medical Society—Notice

**Published:** 1855-05

**Authors:** 


The Iowa Mecical and Chirugical Society

Will hold its next Annual Meeting at Keokuk, on Wednesday, the 14th of June, 1855, commencing at 10 o'clock, A. M.

J. HOWES, R. S. Sec'y.

Medical men, who have interesting cases in Surgery, will please report them to the undersigned, Chairman of the Committee on Surgery.

Please address J. C. HUGHES, M. D.,

Chairman Com. on Surgery,

April 27th 1855. Keokuk, Iowa.

We hope to see a large assemblage of medical men of the State at our State Medical Convention, to be holden in this place in June next. We are glad to perceive that in other States, there is a growing interest in these State organizations, and we are the more and better pleased that the transactions are so fruitful in their results to the cause of medical science. When we receive these printed reports of their doings, we infer that the profession of those States is in the line of progress, and that the members are determined not to be behind the age of science. It is a true test of an emulous spirit, and an evidence of their prominence when these reports come laden with an assemblage of facts. Those States without an organization of the kind are looked upon as under some dark cloud which obscures them from the sun light of science, for we hear nought of them, and per-consequence conclude they are dragging behind and far in the rear. Such is the natural and inevitable conclusion.

Since the organization of our State Society, several reports have been made of our doings, which have been extensively noticed in the journals, and in a manner highly flattering to the profession of this young State. Recently the "American Journal of Medical Science," of Philadelphia, noticed them at length, preceded by some pertinent remarks upon the advantages of such organizations.

We say to our friends come up to the next meeting laden with the fruits of your labors of the past year and contribute to the richness of the repast which we hope is in store for us all. Come one and all and renew to our medical friends abroad the assurances of our continued efforts in the great work of improvement, and that here west of the Mississippi, medical science is being cultivated with an ardor and devotion which will entitle us to a prominence and conspicuity in proportion to the number and value of our contributions to its advancement.

Let us then come together animated by considerations of respect for our exalted calling, compare opinions and give to each other the results of our experience. It is said there is strength in union, but how can we be united unless we meet one another and by that means arrive at a knowledge of each other. There is zeal among the members of the profession of our State, talent too of a high order, and acquirements extensive and liberal. Come then and swell the society in numbers, and give it character by substantial contributions to its transactions.

